# A Binocular Color Line-Scanning Stereo Vision System for Heavy Rail Surface Detection and Correction Method of Motion Distortion

**DOI:** 10.3390/jimaging10060144

**Published:** 2024-06-13

**Authors:** Chao Wang, Weixi Luo, Menghui Niu, Jiqiang Li, Kechen Song

**Affiliations:** 1 School of Transportation, Ludong University, Yantai 264025, China; ljq7436@ldu.edu.cn; 2 School of Mechanical Engineering & Automation, Northeastern University, Shenyang 110819, China; luoweixineu@stumail.neu.edu.cn; 3 Beijing Institute of Control and Electronics Technology, Beijing 100045, China; numhui@163.com

**Keywords:** heavy rail surface defects, line-scanning stereo vision, stereo matching, motion distortion correction, cubature Kalman filter (CKF)

## Abstract

Thanks to the line-scanning camera, the measurement method based on line-scanning stereo vision has high optical accuracy, data transmission efficiency, and a wide field of vision. It is more suitable for continuous operation and high-speed transmission of industrial product detection sites. However, the one-dimensional imaging characteristics of the line-scanning camera cause motion distortion during image data acquisition, which directly affects the accuracy of detection. Effectively reducing the influence of motion distortion is the primary problem to ensure detection accuracy. To obtain the two-dimensional color image and three-dimensional contour data of the heavy rail surface at the same time, a binocular color line-scanning stereo vision system is designed to collect the heavy rail surface data combined with the bright field illumination of the symmetrical linear light source. Aiming at the image motion distortion caused by system installation error and collaborative acquisition frame rate mismatch, this paper uses the checkerboard target and two-step cubature Kalman filter algorithm to solve the nonlinear parameters in the motion distortion model, estimate the real motion, and correct the image information. The experiments show that the accuracy of the data contained in the image is improved by 57.3% after correction.

## 1. Introduction

For high-speed rail transportation, the quality of heavy rail is a key factor to ensure the safety of train operations. As an important means of controlling the quality of heavy rail products, the surface quality detection of heavy rail directly affects the economic benefits of enterprises and the safety of railway transportation. Different from common industrial product detection and in-service track detection [1,2], the surface quality detection of heavy rail in production lines has the following problems and challenges: harsh working conditions, the complex surface background of rolling heavy rail, scattered and random defects, high production and transmission speed, and continuous work. The existing surface defect detection methods for heavy rail production lines mainly include manual detection methods, traditional non-destructive detection methods, and detection methods based on machine vision. Among them, machine vision technology, with its advantages of non-contact and strong adaptability, can effectively meet the requirements of on-site high-speed production quality inspection. Compared to other detection methods, machine vision has higher flexibility and automatic deployment characteristics and is more adaptable to the usage environment.

As early as the 1990s, machine vision technology had been applied to the surface defect detection process in industrial production. In 1990, Piironen et al. introduced a prototype of an automatic visual online metal strip detection system [3]. In 2013, Song et al. [4] presented an automatic recognition method for hot-rolled steel strip surface defects affected by the feature variations of intra-class, illumination, and grayscale. Yang et al. investigated the online fault detection technique based on machine vision for conveyor belts [5]. Wang et al. [6] proposed an automatic detection method for rail fastener defects based on machine vision. In 2018, Dong et al. proposed an automatic defect detection network for surface defect detection, which achieved high-precision defect detection on data sets such as steel strips, ceramic tiles, and road surface defects [7]. To optimize the efficiency of rail defect detection, Cheng et al. [8] proposed a detection algorithm for a Faster Region-based Convolutional Neural Network (Faster R-CNN) based on machine vision in 2020. Song et al. [9] proposed a significance propagation algorithm (MCITF) based on multiple constraints and improved texture features to solve the complex variation of strip surface defects and the similarity of defects between classes. Zhang et al. [10] proposed a unified method to detect both the common and rare defects on the surface of aluminum profiles, which develops an attention module to promote the accuracy of the common and rare defects by providing PMs. In 2021, Zhou [11] studied a rail defect detection system based on machine vision to identify the rail track areas that require polishing. Guo et al. [12] used the Mask R-CNN network to detect defects on the rail surface and conduct semantic segmentation of defect areas. In 2022, Ma et al. [13] proposed a novel one-shot unsupervised domain adaptation framework for rail surface defect segmentation under different service times and natural conditions, which effectively improved the robustness of the model to distribution differences. Sun et al. [14] proposed an unsupervised defect detection system for aluminum plate inspection using a combination of bright-field and dark-field illumination in 2023.

At present, the 3D measurement methods in heavy rail detection research mainly include line-structured light triangulation measurement and stereo vision measurement. Among them, the stereo vision system based on linear array cameras benefits from the advantages of a large field of view, ultra-high resolution, and high acquisition speed [15]. It has higher optical accuracy, field of view range, and data transmission efficiency and is more suitable for continuous operations and high-speed transmission of industrial product inspection sites. It can also collect corresponding color and texture information while obtaining three-dimensional data [16,17,18]. Therefore, the scheme based on linear array stereo vision is also more conducive to the acquisition of 3D data of the heavy rail surface in the production line.

In 2009, Huke et al. [19] first proposed the three-dimensional measurement method using structured light based on a single line-scanning camera. In 2015, Lilienblum [20] proposed a linear scanning three-dimensional measurement method based on the intersection measurement of two line-scanning cameras, gradually improving the principal basis and equipment model of linear array stereo vision. On this basis, Niu et al. proposed an unsupervised stereo saliency detection method based on a binocular line scanning system, which provides an effective method for locating rail surface defects [21]. In 2021, Wu et al. [22] proposed a linear laser scanning measurement method to complete the 3D scanning of a freeform structure. However, in the application of stereo vision, it is possible to obtain incorrect detection results by directly using the acquired image data. This is because of the one-dimensional imaging feature of the line-scanning camera. When the scanning plane of the visual sensor is not perpendicular to the motion transmission direction, there will be oblique deformation in the scanned image, which will affect the authenticity of the description of the surface texture of the heavy rail.

The binocular color linear array stereo vision system studied in this paper adopts the measurement principle of coplanar intersection. Different from the known measurement methods of different plane intersections, coplanar intersection measurement has greater flexibility and higher accuracy in obtaining the surface profile information of the measured object [23]. The visual field planes of the two cameras coincide, which can ensure the synchronous imaging of measurement points in the space between the two cameras. The depth calculation can be carried out only by searching the corresponding points in the same frame, which is suitable for obtaining three-dimensional point clouds of the surface with weak texture in various poses. At the same time, the coplanar intersection measurement avoids introducing additional motion information and the limit constraints in the matching process, which greatly improves the speed and accuracy of registration, and reduces the computational cost [22,24,25]. The motion distortion in this method is mainly due to the installation error between the camera and the transmission motion device, the vibration in the transmission process, and the image stretching or compression caused by frame rate mismatch between image acquisition and rotary encoder. The huge motion distortion not only generates erroneous contour information, increases the false alarm rate, and leads to abnormal detection but also affects the screening, classification, and localization of suspected defect areas in the actual detection process due to inaccurate information transmission [26]. To reduce the influence of the above factors, a checkerboard target with coordinate information is used to estimate the actual motion situation and correct the final image information. It can not only guide the device installation but also compensate for the impact of motion distortion. The double-step cubature Kalman filter algorithm is used to iteratively solve the nonlinear parameters of the motion distortion model. The effectiveness of the correction algorithm is verified through experiments.

## 2. Hardware Acquisition System

The binocular color line-scanning stereo vision system consists of a binocular color line-scanning camera, an illumination system, and an experimental transmission motion platform.

### 2.1. Binocular Color Line-Scanning Camera

The hardware of the binocular color line-scanning system mainly includes a line-scanning camera unit, a binocular integration unit, and a data acquisition and transmission cooperative control unit. Compared with the area-scanning camera, the sensor units of the line-scanning camera are mainly concentrated in the length direction, and the number can easily reach several thousand, while the width direction is only a few rows. This allows line-scanning CCD cameras to have a larger field of view and higher resolution in the lengthwise direction than aera-scanning cameras [1]. In addition, the reduction in the overall number of light-sensitive units allows line-scanning cameras to achieve higher scanning frequencies. In practical applications, line-scanning cameras can be used to continuously image products to be detected using scanning stitching. Therefore, line-scanning cameras are widely used in the detection of industrial products with continuous and uniform motion, such as metals, plastics, paper, and cloth fabrics.

**Figure 1:**
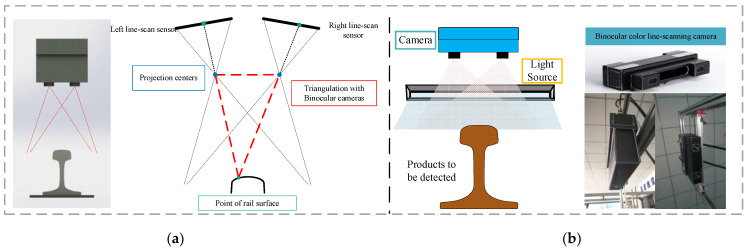
Binocular color line-scanning camera system: (**a**) Structure schematic diagram; (**b**)Picture of the actual system.

Binocular color line-scanning stereo vision system is built based on binocular vision measurement, and the triangulation principle is used to obtain RGB color images and calculate their corresponding 3D depth information, as shown in Figure 1. The color line-scanning camera system used in this paper is mainly based on the 3DPIXA camera of Chromasens, which is mainly oriented to high-speed 3D measurement applications, such as food production detection, industrial parts, and natural target image reconstruction. It uses a trilinear CCD line sensor (RGB) that interacts with the PC through CammeraLink. In addition to its built-in 3D_API, it also supports industrial image processing software such as HALCON (MVTec) for various related subsequent vision application development, as shown in Figure 1. The optical resolution of the camera is up to “70 µm/pixels”, and its maximum acquisition speed is up to “1.4 m/s” with a maximum frame rate of “21 kHz”. In addition, the line-scanning camera has 7142 ultra-high resolution pixels in each line to meet the RGB three-channel color information capture. The specific parameters of the camera are shown in Table 1.

### 2.2. Lighting System Selection and Layout

**Figure 2:**
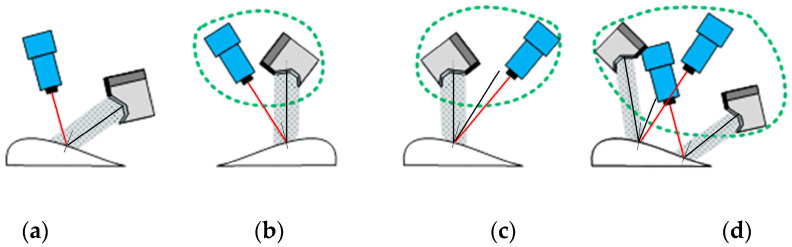
Various lighting layout methods and applicability camera types: (**a**) Bright field line scan; (**b**) Compact bright field surface scan; (**c**) Bright dark field surface scan; (**d**) Bright field/dark field surface scan.

Inappropriate lighting can cause a lot of problems. For example, blotches and overexposure can hide a lot of important information, and shadows can cause false detection of edges. The reduced signal-to-noise ratio, as well as non-uniform illumination, can lead to difficulties in selecting the threshold for image processing. The factors to consider in choosing the optimal lighting scheme include light intensity, polarization, uniformity, direction, size and shape of the light source, diffuse or linear, background, etc., and the optical characteristics of the test objects (color, smoothness, etc.), working distance, object size, luminescence, etc., as shown in Figure 2.

Table 2 shows the main properties of halogen lamps, fluorescent lamps, and LED light sources. As can be seen from the table, LED light source has high efficiency, small size, less heat, low power consumption, stable luminescence, and long life (red LED life can reach 100,000 h, while other colors can also reach 30,000 h), and can be designed into light sources of different shapes and lighting modes through different combinations, such as ring lamp, dome lamp, coaxial light source, strip lamp, etc.

If a black-and-white camera is used, there is no special requirement for the color selection of the measured object, and a red LED is the most appropriate choice. Generally, CCD is not sensitive to purple and blue light, and CCD without coating is the most sensitive in the near-infrared region. If color imaging is performed, a white light source must be used.

Whether the final image acquisition effect can meet the requirements mainly depends on the layout relationship between the lighting system and the CCD camera. Different layout methods, such as linear light source, planar scattering light source, line-scanning CCD camera, area-scanning CCD camera, and mixed layout, have different effects on the resolution and contrast of the collected image. Several common layouts are shown in Figure 2. For binocular line-scanning cameras with ultra-high resolution, the small aperture setting is usually used to reduce lens distortion, minimize assembly errors, and expand the depth of field range.

**Figure 3:**
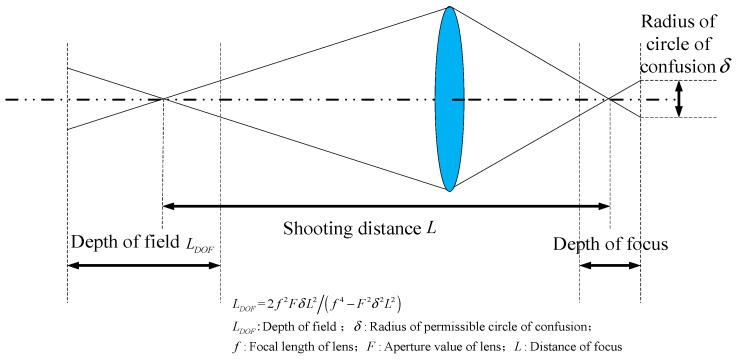
Camera imaging internal related parameters.

**Figure 4:**
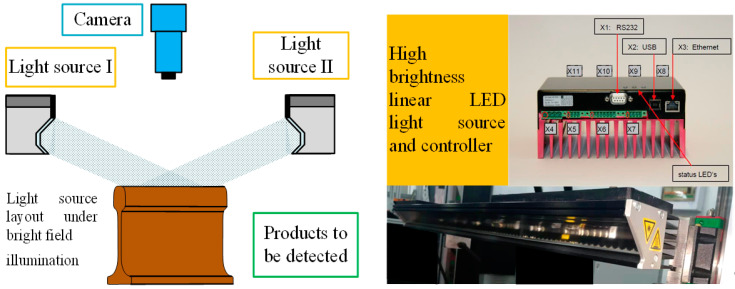
Symmetrical linear LED light source layout.

The binocular color line-scanning stereo vision system covered in this paper uses a bright-field illumination scheme. As shown in Figure 3, the depth of field when the camera is shooting varies with the focal length, aperture value, and distance. When the aperture becomes smaller, the front and back of the subject become clearer, but the overall picture becomes darker due to the amount of light intake. To capture clearer imaging results, it is necessary to use a small aperture for shooting while increasing the amount of light input. Therefore, a bright field lighting scheme is necessary. Because a single light source illumination cannot meet the demand, the symmetrical linear LED light source layout, as shown in Figure 4, is adopted.

### 2.3. Experimental Transmission Motion Platform

**Figure 5:**
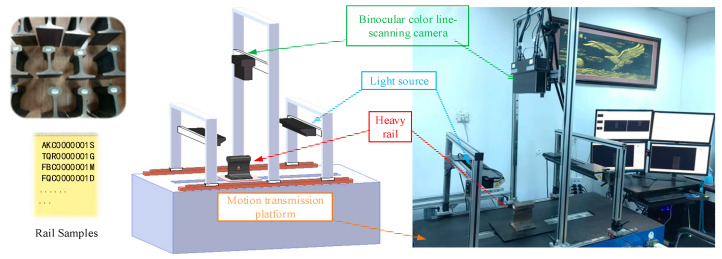
Experimental platform device.

**Figure 6:**
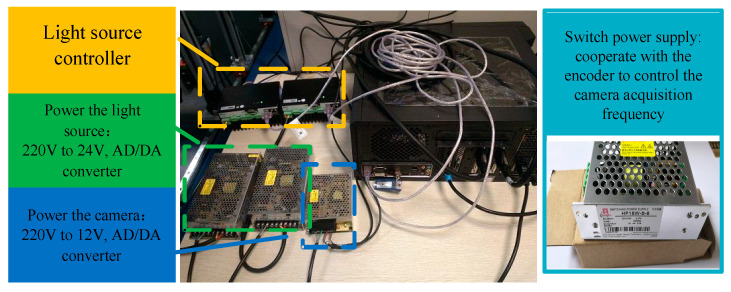
Corona II light source control system.

In order to meet the experimental requirements of the line-scanning stereo vision system, the target object to be detected needs to have relative motion with the camera and a relatively stable proportional coordination relationship between its motion speed and the camera acquisition frequency under general conditions. In this paper, the motion experimental platform device shown in Figure 5 is used to carry out the relevant research on the line-scanning stereo vision system, and the device composition is shown in Table 3. The scanning system adopts the 3DPIXA binocular color line-scanning camera made by Germany Chromasens, model 3DPIXA-Dual70μm. The supporting image acquisition card is the microEnable series acquisition card of the German Silicon Software company, and the model is microEnable ⅣAD4-CL. The model of the linear LED light source is Corona Ⅱ. The XLC4 light source controller is used to adjust the brightness of the light source by controlling the current, and its adjustable range is 200–1800 mA. The power converter device is shown in Figure 6. The motion platform adopts an incremental encoder, model Sendix Base KIS40. The overall acquisition and control system relies on a desktop PC with an Intel(R) Core(TM) i7-7700 CPU.

The software mainly includes image acquisition and control software, light source adjustment and control software, camera parameter configuration software, and CS-3D-Viewer V3.2.0 development software based on 3DPIXA, as shown in Table 4.

## 3. Principles of Triangulation and Stereo Matching

### 3.1. Triangulation Principle of Binocular Line-Scanning Camera

**Figure 7:**
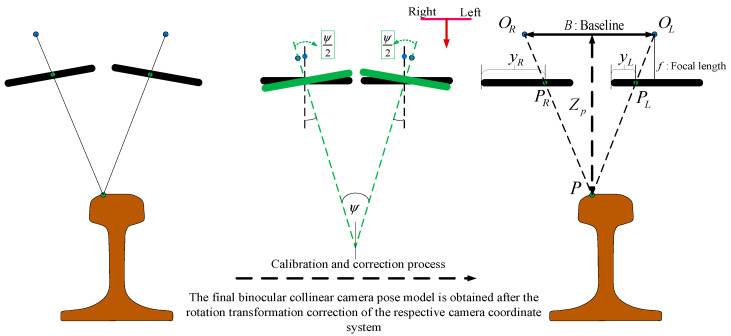
Coplanar intersection measurement with binocular line scanning camera.

Triangulation is an effective measurement method in the field of machine vision. The improved triangulation methods achieve better point and stereo triangulation results [27,28], which are then used for on-machine measurement technology for complex surfaces [29]. The binocular linear-scanning camera in this paper adopts the principle of coplanar intersection measurement. When the field-of-view planes of the two line-scanning cameras coincide exactly, and after correction process, it can be simplified to a coplanar geometric model as shown in Figure 7. Where the optical centers of the two cameras after correction are $O_{R}$ and $O_{L}$, respectively, which are rotated to the back of the imaging plane to simplify the description of the principle of triangulation. P is the point to be measured in space, $P_{L}$ and $P_{R}$ are the imaging points at the spatial point P on the left and right camera planes. B is the baseline distance between the optical centers of the two cameras, and the focal length of the two cameras is f. $Z_{P}$ is the depth distance from the spatial point to the camera coordinate system. Let $P_{RL}=B-(y_{R}-y_{L})$, then according to the principle of triangle similarity, it can be concluded that

(1) $$\frac{P_{RL}}{B}=\frac{Z_{p}-f}{Z_{p}}$$

**Figure 8:**
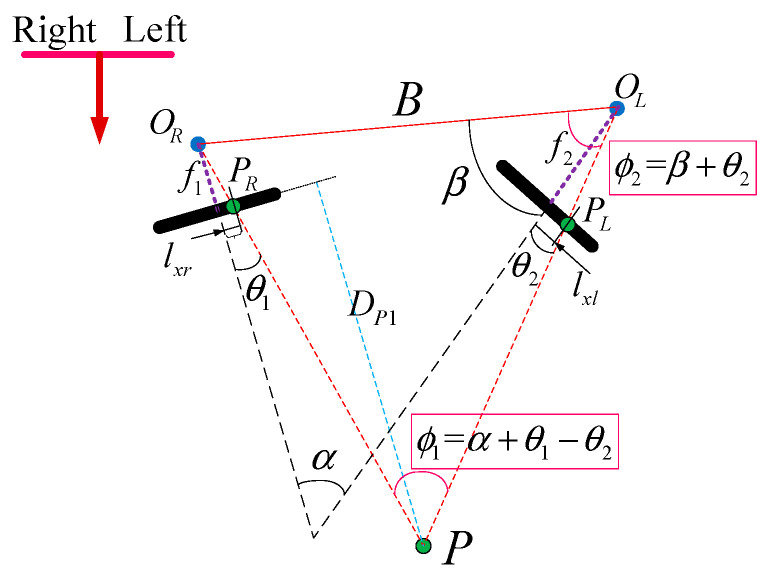
Simplified model of binocular triangulation.

Let $d=(y_{R}-y_{L})$, then the depth distance from the spatial point P to the camera baseline B is

(2) $$Z_{p}=\frac{f\cdot B}{d}$$

where d is the position deviation of pixels imaged under two cameras of the same scene, which is the disparity in binocular matching. However, in general, the intrinsic parameters of the cameras are not the same, especially when the camera models are not consistent or there is a gap in the focal length adjustment, the imaging model is not applicable. The real simplified model is shown in Figure 8.

Where the optical centers of the two cameras are $O_{R}$ and $O_{L}$, respectively. P is the position point in space, and $P_{L}$ and $P_{R}$ are their corresponding imaging points. B is the baseline distance between the two cameras, the focal lengths of the two cameras are $f_{1}$ and $f_{2}$, respectively, and $D_{P1}$ is the depth distance from the spatial point to the imaging plane of the right camera coordinate system. In addition, α is the angle between the optical axes of the two cameras, β is the angle between the optical axis of the left camera and the baseline. $\theta_{1}$ is the angle between the projection line of the spatial point on the right camera and the optical axis of the right camera, and $\theta_{2}$ is the angle between the projection line of the spatial point on the left camera and the optical axis of the left camera. $l_{xr}$ is the distance between the projection point of the right camera and the principal point of the right camera, and $l_{xl}$ is the distance between the projection point of the left camera and the principal point of the left camera. According to the geometric properties of the triangle and the formula for the sine area of the triangle, there is

(3) $$S_{\Delta PO_{R}O_{L}}=\frac{1}{2}l_{PO_{R}}\cdot l_{PO_{L}}\cdot \sin(\phi_{1})=\frac{1}{2}B\cdot l_{PO_{L}}\cdot \sin(\phi_{2})$$

where, $\phi_{1}$ is the angle between the projection line of the spatial point in the left camera and the baseline, and $\phi_{2}$ is the angle between the projection line of the spatial point in the left and right cameras. $l_{PO_{R}}$ and $l_{PO_{L}}$ are the distances from the spatial point P to the optical centers of the left and right cameras, respectively. The above equations can be simplified as follows:

(4) $$l_{PO_{R}}=B\frac{\sin(\phi_{2})}{\sin(\phi_{1})}$$

It can be obtained that the distance from the spatial point to the imaging plane of the right camera is

(5) $$D_{P1}=\left(l_{PO_{R}}-\frac{f_{1}}{\sin(\theta_{1})}\right)\cdot \cos(\theta_{1})$$

In summary, it can be obtained that

(6) $$D_{P1}=\left(B\frac{\sin(\beta +\theta_{2})}{\sin(\alpha +\theta_{1}-\theta_{2})}-\frac{f_{1}}{\sin(\theta_{1})}\right)\cdot \cos(\theta_{1})$$

where

(7) $$\{\begin{array}{c} \theta_{1}=\arctan(\frac{l_{xr}}{f_{1}})\\ \theta_{2}=\arctan(\frac{l_{xl}}{f_{2}}) \end{array}$$

### 3.2. Binocular Vision Stereo Matching

**Figure 9:**
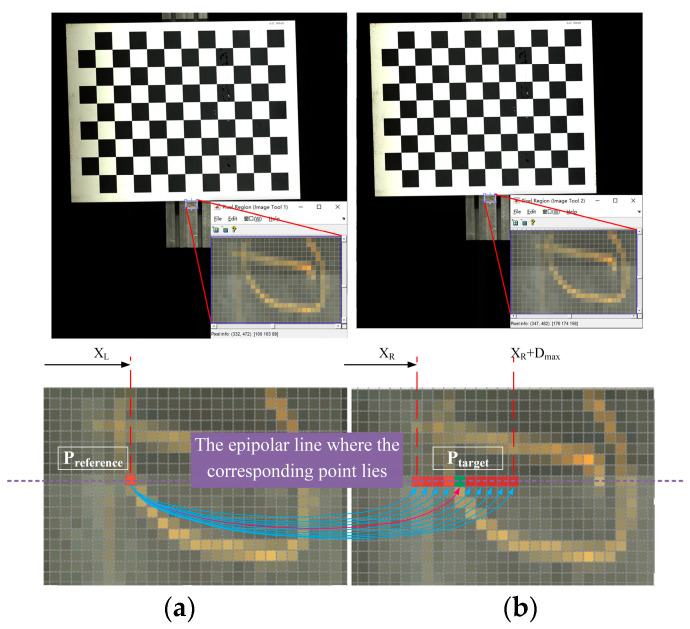
Schematic diagram of binocular stereo matching: (**a**) Left camera correction image; (**b**) Right camera correction image.

Stereo matching is the key part of the stereo vision reconstruction, which mainly restores the spatial information of the 3D world through multiple images [30,31,32]. The purpose is to find the same point in two or more view images and then obtain the disparity result for depth estimation. As shown in Figure 9, after the epipolar rectification, the reference point in the left view is $P_{reference}$, and the optimal target point position $P_{target}$ is found by searching the homonymy points on the epipolar line of the right view and within the disparity range $D_{max}$.

The matching algorithm in stereo reconstruction involved in this paper is mainly based on the semi-supervised global matching algorithm SGBM (Semi-Global Block Matching) [33]. The initial disparity map is constructed by calculating and selecting the disparity corresponding to each pixel, and the related global energy function is established. By solving this minimization energy function, the disparity result corresponding to each pixel is optimized. The minimization energy function equation is as follows:

(8) $$E(D)=\sum_{p}\left(\mathrm{Cos}t(p,D_{p})+\sum_{q\in N_{p}}\lambda_{1}\cdot I_{1}\left[\left|D_{p}-D_{q}\right|=1\right]+\sum_{q\in N_{p}}\lambda_{2}\cdot I_{2}\left[\left|D_{p}-D_{q}\right|>1\right]\right)\;$$

where $Cost\left(p,D_{p}\right)$ is the matching cost. $\sum \lambda_{1}\cdot I_{1}(\cdot )$ and $\sum \lambda_{2}\cdot I_{2}(\cdot )$ are the neighborhood disparity cost penalty terms. $I_{1}(\cdot )$ and $I_{2}(\cdot )$ are the indicator functions (returns “1” if true, in parentheses, otherwise it will return “0”). D is the current corresponding overall disparity map, $D_{p}$ is the disparity result corresponding to point p, and $D_{q}$ is the disparity result corresponding to point q. $q\in N_{p}$ is the neighborhood of point p, $\lambda_{1}$ is the coefficient of the penalty term of $I_{1}\left(\cdot \right)$ with a disparity difference of 1 in the neighborhood, and $\lambda_{2}$ is the coefficient of the penalty term of $I_{2}\left(\cdot \right)$ with disparity difference greater than 1 in the neighborhood.

In the process of calculating the initial disparity cost, the sliding window is usually used. The smaller the matching cost calculation window is, the more noisy the disparity map is. On the contrary, the larger the window setting, the smoother the disparity map. However, a window too large can easily lead to over-smoothing and increase the probability of mismatching, as well as more void regions with no values in the disparity map. The smoothness of the final disparity map result is controlled by using the penalty coefficient and penalty term. The larger the $\lambda_{2}$, the smoother the disparity map.

In summary, according to the principle of triangulation and epipolar rectification, and combined with the SGBM stereo matching algorithm, the disparity map of the left and right cameras is obtained. Finally, the intrinsic and extrinsic parameters of each camera and between cameras of the binocular line-scanning system were obtained by calibration and other methods, and the final depth map was obtained through the baseline distance and disparity. In particular, the left-right consistency detection error of the SGBM algorithm is “10”, and the matching disparity range is “−165,+165”. Based on the above conditions, the binocular line-scanning system can provide high-precision depth information with a resolution of “14 µm” in the range of “52 mm”.

## 4. Motion Distortion Correction

### 4.1. Motion Distortion

**Figure 10:**
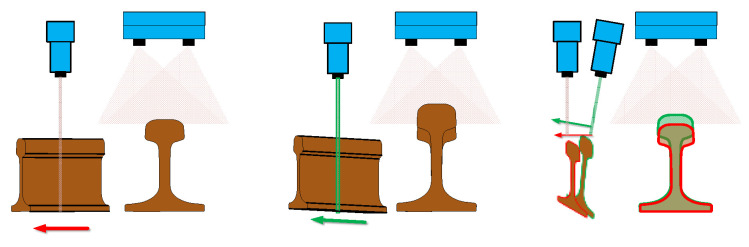
Schematic diagram of motion distortion caused by installation Error.

**Figure 11:**
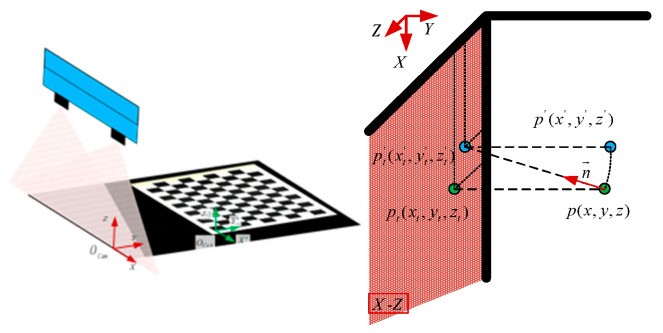
Correspondence between real points $p_{t}^{\prime }(x_{t}^{\prime },y_{t}^{\prime },z_{t}^{\prime })$ and image mapping points p(x,y,z) caused by installation error.

The main reasons for motion distortion are the installation error between the camera and the transmission motion device and the vibration during transmission, as shown in Figure 10. Firstly, when the angle between the camera’s view plane and the motion direction exists, due to the influence of the scanning imaging factors of the linear-scanning camera, the image not only exists stretching or compression deformation in the direction perpendicular to the field of view plane but also produces a displacement along the direction of the view plane. Secondly, although the real-time reconstructed contour of the object to be measured is robust to vibration, in the process of motion stitching, the motion relationship between the object to be measured and the camera is distorted due to vibration influence, resulting in errors in the final stitched overall contour along the motion direction. To reduce the influence of the above factors, the checkerboard target with coordinate information is used to estimate the actual motion situation and correct the final image information. As shown in Figure 11, the X−Z plane is the scanning plane of the camera’s field of view, the point p(x,y,z) in space is a point on the target object, $\vec{n}=(n_{1},n_{2},n_{3})$ is the actual direction of motion transmission. $p_{t}^{\prime }(x_{t}^{\prime },y_{t}^{\prime },z_{t}^{\prime })$ is the vertical projection point of point p(x,y,z) on the camera and p′(x′,y′,z′) is the real projection point of point p(x,y,z) on the camera image along the motion direction, p′(x′,y′,z′) is the position of the corresponding spatial point displayed in the camera imaging result.

When the relationship between the acquisition frequency of the camera and the true motion velocity is not changed, it is known that the distance $L_{t}$ from point p to point $p\prime_{t}$ is equal to the distance $L\prime_{t}$ from point $p\prime_{t}$ to point p′. Therefore, the following relationship can be obtained:

(9) $$\{\begin{array}{c} x^{\prime }=x-\frac{y}{n_{2}}\cdot n_{1}\\ y^{\prime }=\frac{y}{n_{2}}\\ z^{\prime }=z-\frac{y}{n_{2}}\cdot n_{3} \end{array}$$

The relationship between the camera and the transmission direction $\vec{n}=(n_{1},n_{2},n_{3})$ can be obtained by using the known relative position coordinate information of the spatial point on the calibration board. In the image coordinate system $O_{Cam}\left(X,Y,Z\right)$ shown in the figure above, the position of the spatial point is p(x,y,z). In the calibration board coordinate system $O\prime_{Cab}\left(X\prime ,Y\prime ,Z\prime \right)$, the coordinate position of the corresponding space point is $p_{c}\left(x_{c},y_{c},z_{c}\right)$. The relationship between the calibration board coordinate system and the image scanning coordinate system can be expressed as follows:

(10) $$p(x,y,z)=R\cdot p_{c}(x_{c},y_{c},z_{c})+T$$

where R and T are the rotation and translation relations between the two coordinate systems, respectively. The above equation is expressed in the coordinate form as

(11) $$\left[\begin{array}{c} x\\ y\\ z \end{array}\right]=\left[\begin{array}{ccc} r_{11} & r_{12} & r_{13}\\ r_{21} & r_{22} & r_{23}\\ r_{31} & r_{32} & r_{33} \end{array}\right]\cdot \left[\begin{array}{c} x_{c}\\ y_{c}\\ z_{c} \end{array}\right]+\left[\begin{array}{c} t_{1}\\ t_{2}\\ t_{3} \end{array}\right]$$

To sum up, it can be concluded that

(12) $$\left\{ \begin{array}{l} n_{2}\cdot y^{\prime }=r_{21}\cdot x_{c}+r_{22}\cdot y_{c}+r_{23}\cdot z_{c}+t_{2}\\ r_{21}^{2}+r_{22}^{2}+r_{23}^{2}=1 \end{array}\right.$$

Solving the above equations yields $n_{2}$, $\left[r_{21},r_{22},r_{23}\right]$ and $t_{2}$. Similarly, the other coordinate correspondences between $p_{c}\left(x_{c},y_{c},z_{c}\right)$ and p′(x′,y′,z′) can be calculated by Equations (13) and (14).

(13) $$\left\{ \begin{array}{l} x^{\prime }=r_{11}\cdot x_{c}+r_{12}\cdot y_{c}+r_{13}\cdot z_{c}+t_{1}-y^{\prime }\cdot n_{1}\\ r_{11}^{2}+r_{12}^{2}+r_{13}^{2}=1 \end{array}\right.$$

and

(14) $$\left\{ \begin{array}{l} z^{\prime }=r_{31}\cdot x_{c}+r_{32}\cdot y_{c}+r_{33}\cdot z_{c}+t_{3}-y^{\prime }\cdot n_{3}\\ r_{31}^{2}+r_{32}^{2}+r_{33}^{2}=1 \end{array}\right.$$

### 4.2. Cubature Kalman Filter for Solving the Relevant Parameters

The above equations can express the relationship between the image acquisition information by the line scanning camera imaging system and the results in real motion. The above equations are integrated as follows:

(15) $$\begin{array}{l} \left[\begin{array}{c} x^{\prime }\\ 0\\ z^{\prime } \end{array}\right]=\left[\begin{array}{ccc} r_{11} & r_{12} & r_{13}\\ r_{21} & r_{22} & r_{23}\\ r_{31} & r_{32} & r_{33} \end{array}\right]\cdot \left[\begin{array}{c} x_{c}\\ y_{c}\\ z_{c} \end{array}\right]+\left[\begin{array}{c} t_{1}\\ t_{2}\\ t_{3} \end{array}\right]-y^{\prime }\cdot \left[\begin{array}{c} n_{1}\\ n_{2}\\ n_{3} \end{array}\right]\\ St:R=\left[\begin{array}{ccc} r_{11} & r_{12} & r_{13}\\ r_{21} & r_{22} & r_{23}\\ r_{31} & r_{32} & r_{33} \end{array}\right],n=\left[\begin{array}{c} n_{1}\\ n_{2}\\ n_{3} \end{array}\right],R\cdot R^{T}=1,n^{T}\cdot n=1, \end{array}$$

In the process of solving, in order to reduce the interference of vibration and other additional factors on image acquisition, this paper uses the cubature Kalman filter to solve the relevant parameters. The specific solution steps are as follows.

The nonlinear system equation and observation equation of motion parameters can be expressed as

(16) $$\begin{array}{l} x_{k+1}^{c}=F\left(x_{k}^{c},\nu_{k}\right)=x_{k}^{c}+\nu (k)\\ y_{k}^{\prime }=H(x_{k+1}^{c},u_{k}^{1},u_{k}^{2},e_{k})=R(x_{k}^{\theta })\cdot u_{k}^{1}+x_{k}^{t}+u_{k}^{2}\cdot x_{k}^{n}+e(k) \end{array}$$

where $u^{1}=\left(x_{c},y_{c},z_{c}\right)$, $u^{2}=y\prime$, and θ is the Euler angle corresponding to the rotation matrix. The parameter vector of parameters to be sought is

(17) $$x^{c}={\left[\theta_{\alpha },\theta_{\beta },\theta_{\gamma },t_{1},t_{2},t_{3},n_{1},n_{2},n_{3}\right]}^{T}=[x^{\theta },x^{t},x^{n}]$$

**Figure 12:**
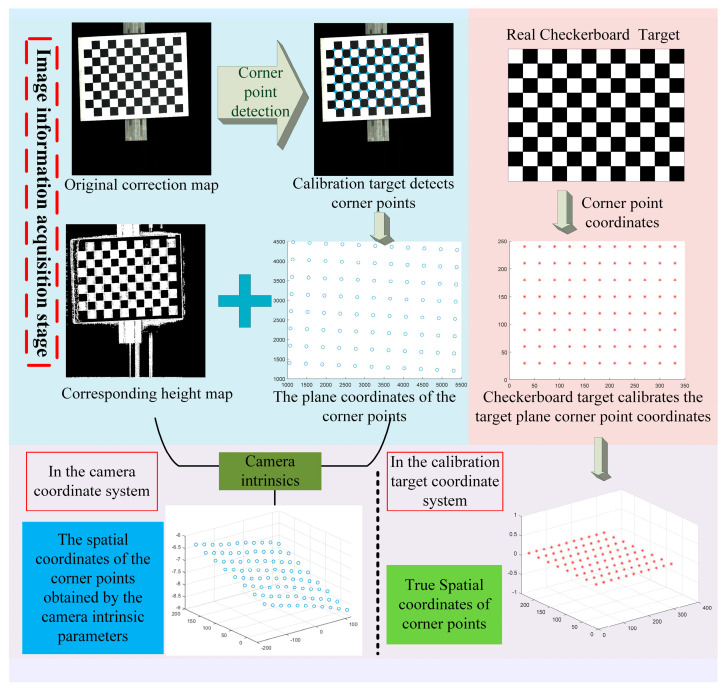
Image information acquisition stage of motion distortion correction process based on cubature Kalman filter.

**Figure 13:**
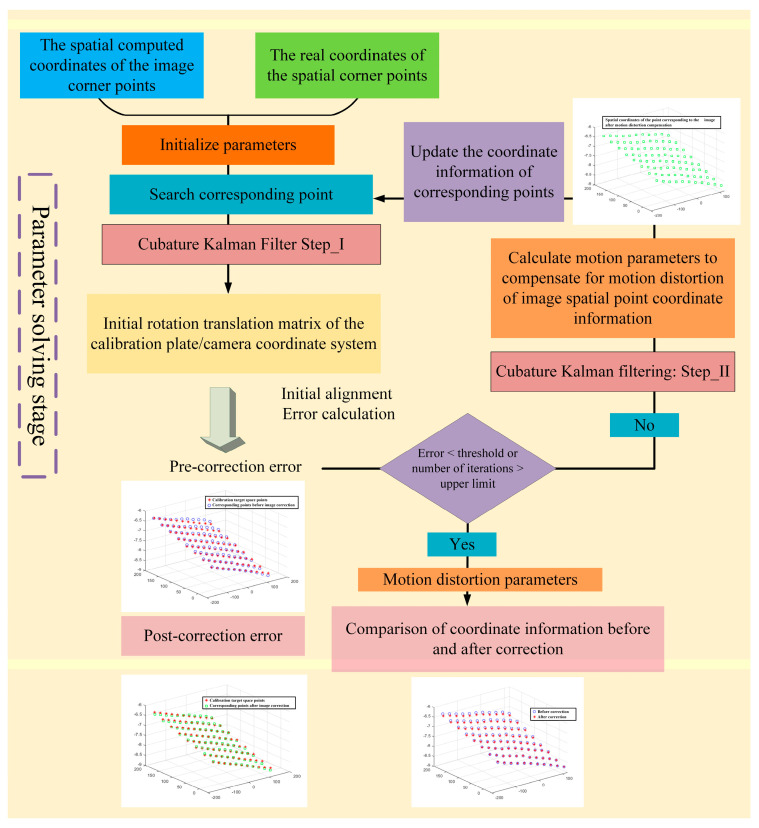
Parameter solving stage of the motion distortion correction process based on cubature Kalman filter.

The flow chart of motion distortion correction based on the cubature Kalman filter is shown in Figure 12 and Figure 13, and its operation steps are shown in Algorithm 1.
**Algorithm 1:** Motion distortion correction based on cubature Kalman filter**Input:** Original calibration target scan map, corresponding depth map, camera intrinsic parameters of binocular line matrix scanning system, parameter information of real calibration target, initialization parameter $x_{0}^{c}$, iteration error threshold Threshold, iteration number upper limit Number. **Step I: Image information acquisition phase:**
1. Corner detection and image coordinate extraction for the original image of the calibration target. 2. Calculate the 3D coordinate information of the image corner points in the camera coordinate system in the previous step based on the binocular camera intrinsic parameters, the corresponding height map and the initial motion transmission correspondence. 3. Based on the information of the real calibration target corner points, set them in the X _Y plane of the calibration target coordinate system with a point spacing of 30mm and a Z coordinate of 0. **Step II: Motion parameter solution stage:**
4. Initialize the parameters $x_{0}^{c}$ in parameters $x_{0}^{n}=[n_{1},n_{2},n_{3}]=[0,1,0]$. 5. Match the point correspondence between the 3D coordinates of the corner points of the image and the real coordinates of the spatial corner points of the calibration target according to the characteristics of the checkerboard graph. 6. Calculate the rotation and translation relationship between the above corresponding points by using the cubature Kalman filter (**Step-I**) to obtain the initial value of $\left[x_{k}^{\theta },x_{k}^{t}]=[\theta_{\alpha },\theta_{\beta },\theta_{\gamma },t_{1},t_{2},t_{3}\right]$ in $x_{k}^{c}$. 7. Re-estimate the root mean square error $Error_{k}$ between the 3D coordinates of the image corner points and the updated coordinates of the spatial corner points in the calibration target after rotating and translating them according to $\left[x_{k}^{\theta },x_{k}^{t}\right]$. 8. If $Error_{k}<=Threshold$ and the number of iterations Iter_times:k<=Number, use the cubature Kalman filter (**Step-II**) to calculate $x_{k}^{n}=[n_{1},n_{2},n_{3}]$ to reduce the Error. Based on the updated $x_{k}^{n}=[n_{1},n_{2},n_{3}]$, the 3D coordinate information of the image corners is updated to compensate and go to step 5. 9. If $Error_{k}<=Threshold$ or k>Number, output the final of k>Number and $x_{k-1}^{n}=[n_{1},n_{2},n_{3}]$. **Output:** Output the final $\left[x_{k}^{\theta },x_{k}^{t}\right]$ and $x_{k-1}^{n}=[n_{1},n_{2},n_{3}]$.

The process of solving the motion state based on volumetric Kalman filtering is as follows:

1. Initialize the state equation and its corresponding covariance matrix $x^{c}=x_{0}^{c},P=P_{0}^{c}$, then initialize the variance in the process model $Q_{k}^{c}=Q_{0}^{c}$.
2. Estimate the predicted states and the predicted state covariance as

(18) $$\begin{array}{l} x_{k+1|k}^{c}=x_{k|k}^{c}\\ P_{k+1|k}=P_{k|k}+Q_{k}^{c} \end{array}$$

3. Then estimate the correspondence points

(19) $$y_{k+1|k}^{\prime }=R(x_{k}^{\theta })\cdot u_{k}^{1}+x_{k}^{t}+u_{k}^{2}\cdot x_{k}^{n}$$

4. Finally, estimate the state and the corresponding covariance matrix

(20) $$\begin{array}{l} x_{k+1|k+1}^{c}=x_{k+1|k}^{c}+K_{k+1}\cdot \left(y_{k+1}^{\prime }-y_{k+1|k}^{\prime }\right)\\ P_{k+1|k+1}=P_{k+1|k}-K_{k+1}\cdot P_{y\prime y\prime ,k+1|k}\cdot K_{k+1}^{T} \end{array}$$

where

(21) $$K_{k+1}=P_{x^{c}y\prime ,k+1|k}\cdot P_{y\prime y\prime ,k+1|k}^{-1}$$

(22) $$P_{x^{c}y\prime ,k+1|k}=Ε\left[(x_{k+1}^{c}-x_{k+1|k}^{c}){\left(y_{k+1}^{\prime }-y_{k+1|k}^{\prime }\right)}^{T}\right]$$

(23) $$P_{y\prime y\prime ,k+1|k}=Ε\left[(y_{k+1}^{\prime }-y_{k+1|k}^{\prime }){\left(y_{k+1}^{\prime }-y_{k+1|k}^{\prime }\right)}^{T}\right]$$

where $K_{k+1}$ is the Kalman gain and $P_{x^{c}y\prime ,k+1|k}$ and $P_{y\prime y\prime ,k+1|k}$ are the covariance matrices. $y_{k+1|k}^{\prime }$, $P_{x^{c}y\prime ,k+1|k}$ and $P_{y\prime y\prime ,k+1|k}$ are obtained by computing the volume points.

## 5. Experimental Results and Analysis

The checkerboard model used in the experiment is GP400-12-9, with an external dimension of 400mm×300mm, a square edge length of 30mm, a pattern array of 12×9, a pattern size of 360×270, an accuracy of ±0.01mm, and a material of float glass. The camera uses a rising edge outer trigger setting, and the cooperative acquisition relationship between the image acquisition frame rate and the rotating encoder pulse frequency is 1:14, and the image gain is 3. The external dual light source is set to 800 mah. The angle installation error between the actual camera view plane and the transmission direction is ≤3°.

**Figure 14:**
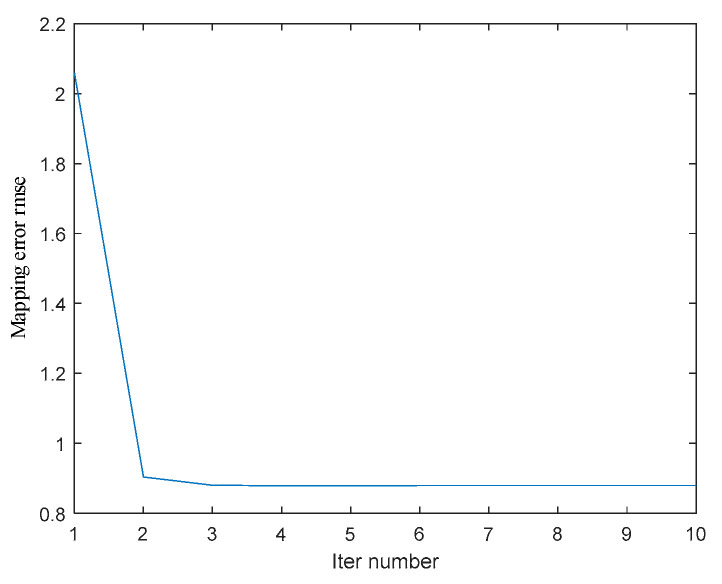
The root mean square error between the corner coordinates of image mapping and the real coordinates is corrected in the iterative process.

**Figure 15:**
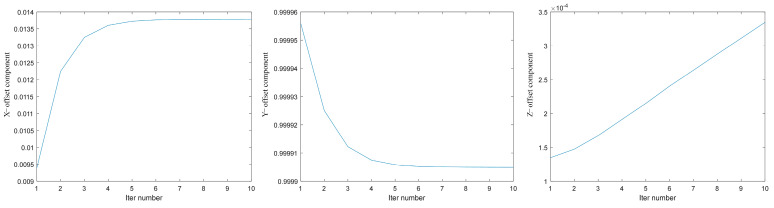
Changes of components of $x_{k}^{n}=[n_{1},n_{2},n_{3}]$ during iteration.

Figure 14 shows the root mean square error (RMSE) of the mapping between the 3D information of the moving image corner points compensated and the coordinates of the real calibration target corner points in each iteration process according to the current motion parameters. The blue curve represents the variation of RMSE with the number of iterations. The figure shows that with the increase of the number of iterations, the results tend to be stable, the RMSE of corner point reprojection is reduced from 2.059 mm to 0.8794 mm, and the data accuracy of the corresponding real coordinate information in the image is improved by 57.3%. Convergence can be achieved after five iterations. The error may be affected by the vibration and noise in motion and the accuracy of depth map information after binocular reconstruction. The variation of each component of $x_{k}^{n}=[n_{1},n_{2},n_{3}]$ during the iteration is shown in Figure 15. The blue curves represent the variations of the offset components in the three directions of x, y, and z with the number of iterations.

**Figure 16:**
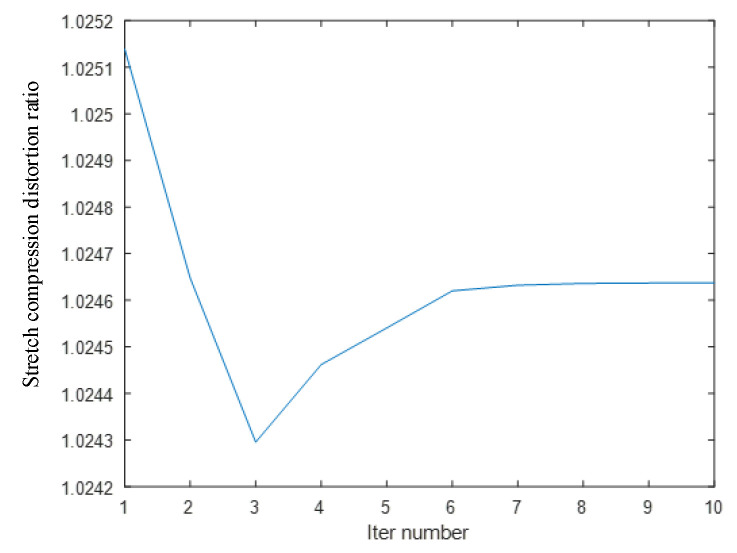
Tensile distortion coefficient during iteration.

**Figure 17:**
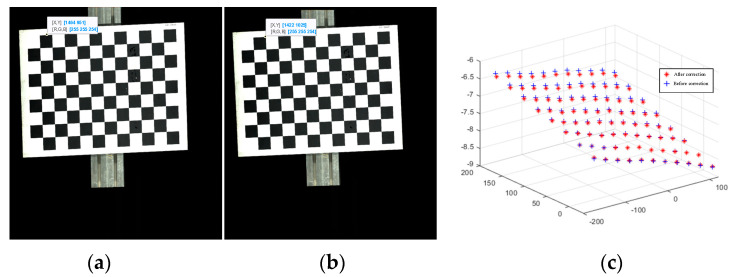
Comparison of image and corner coordinates before and after motion distortion: (**a**) Before correction; (**b**) After correction; (**c**) Coordinates of corner points before and after correction.

**Figure 18:**
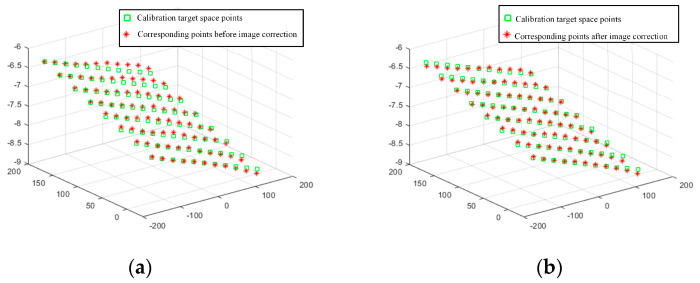
The real corner coordinates are the same as the corner coordinates of the image before correction and the corner coordinates of the image after correction: (**a**) Before correction; (**b**) After correction.

**Figure 19:**
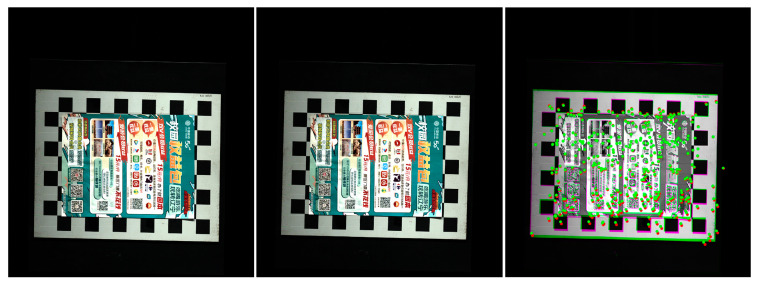
Before and after comparison of corrected images of advertising brochures.

In addition to the offset of the information in the image caused by the installation error, the image stretch or compression caused by the mismatch between the image acquisition frame rate and the frame rate of the rotary encoder is also an important source of image information deviation. Figure 16 shows that in this experiment, there is a stretching distortion effect between the image acquisition frame rate and the rotary encoder frame rate with a distortion ratio of approximately 1.0246:1. The blue curves represent the variation of the tensile distortion coefficient with the number of iterations. The distortion effect is mainly reflected in the normalization process of the motion vector n. When normalizing n, the normalization coefficient is the stretching distortion coefficient. Figure 17 shows the calibration target image before correction and the calibration target image after correction, as well as the 3D information display of their corresponding image corner points in the camera coordinate system. Figure 18 shows the comparison between the real corner point coordinates and the corner point coordinates of the image before and after correction, respectively. The error shows that the corrected image can reflect the spatial position relationship of the target to be detected more realistically and reduces the influence brought by the system installation error and the cooperative acquisition error. Figure 19 shows the before-and-after comparison of the corrected image of the advertising brochure, where the red and green dots indicate the strong correspondence between the original image and the corrected image, respectively.

## 6. Conclusions

In this paper, a binocular color line-scanning stereo vision system is designed, which utilizes the triangulation principle and stereo matching technology to capture high-precision and high-resolution 2D images and 3D contour information of the heavy rail surface. A model is established to address the motion distortion of captured images caused by camera installation errors and collaborative acquisition mismatch. The parameters in the nonlinear model are iteratively solved by a checkerboard target and the double-step cubature Kalman filter algorithm. The experiments prove that the RMSE decreases from the original 2.059 mm to 0.8794 mm, and the data accuracy of the corrected coordinate information in the image is improved by 57.3%.

## Figures and Tables

**Table 1:** Camera parameters of 3DPIXA.

Performance	Parameter
Optical resolution	70 μm/pixel
Field of view	500 mm
Pixels	7142
Pixel unit	10 × 10 μm
Height resolution	14 μm
Depth of field	52 mm
Distance of working surface	796.9 mm
Detection speed	1480 mm/s

**Table 2:** Performance comparison of common light sources.

Performance	Halogen	Fluorescent	LED Light Source
Lifespan (hours)	5000–7000	5000–7000	60,000–100,000
Brightness level	bright	brighter	High brightness (multiple LEDs)
Response speed	slow	slow	fast
Characteristic	High heat generation, almost no change in brightness and color temperature, cheap price.	Less heat generation, good diffusivity, suitable for large area uniform irradiation, and cheap.	Less heat generation, the wavelength can be selected according to the application, the shape is convenient to make, the operation cost is low, and the power consumption is low.

**Table 3:** Hardware composition and function of the experimental sports platform.

Hardware	Quantity	Function
3DPIXA camera	1	Image acquisition
Corona linear LED light source	2	Provide light and increase the amount of light intake
XLC4 controller	2	Regulate the brightness of the light source
220 V to 24 V, AD/DA converter	2	Power the light source
220 V to 12 V, AD/DA converter	1	Power the camera
MicroEnable Image Capture Card	2	Capture digitized video image information and store it
KIS40 encoder	1	Collaborative control the relationship between transmission speed and camera acquisition frequency
Transmission motion platform	1	Realize the relative movement of the product to be detected and the camera

**Table 4:** Software and function of experimental sports platform.

Software	Features
XLC4Commander	Connect XLC4 to PC to control the brightness of the linear LED light source
Camera Setup Tool (CST)	Configure camera-related preset parameters
MicroDisplay	Real-time display of image acquisition
CS-3D-Viewer	Provide intrinsic parameter conversion of the camera and subsequent development SDK

## Data Availability

The raw data supporting the conclusions of this article are not publicly available at this time but will be made available by the authors on request.
